# Adaptation and Preadaptation of *Salmonella enterica* to Bile

**DOI:** 10.1371/journal.pgen.1002459

**Published:** 2012-01-19

**Authors:** Sara B. Hernández, Ignacio Cota, Adrien Ducret, Laurent Aussel, Josep Casadesús

**Affiliations:** 1 Departamento de Genética, Universidad de Sevilla, Sevilla, Spain; 2 Laboratoire de Chimie Bactérienne, CNRS-UPR 9043, Aix-Marseille University, Marseille, France; Stanford University School of Medicine, United States of America

## Abstract

Bile possesses antibacterial activity because bile salts disrupt membranes, denature proteins, and damage DNA. This study describes mechanisms employed by the bacterium *Salmonella enterica* to survive bile. Sublethal concentrations of the bile salt sodium deoxycholate (DOC) adapt *Salmonella* to survive lethal concentrations of bile. Adaptation seems to be associated to multiple changes in gene expression, which include upregulation of the RpoS-dependent general stress response and other stress responses. The crucial role of the general stress response in adaptation to bile is supported by the observation that RpoS^−^ mutants are bile-sensitive. While adaptation to bile involves a response by the bacterial population, individual cells can become bile-resistant without adaptation: plating of a non-adapted *S. enterica* culture on medium containing a lethal concentration of bile yields bile-resistant colonies at frequencies between 10^−6^ and 10^−7^ per cell and generation. Fluctuation analysis indicates that such colonies derive from bile-resistant cells present in the previous culture. A fraction of such isolates are stable, indicating that bile resistance can be acquired by mutation. Full genome sequencing of bile-resistant mutants shows that alteration of the lipopolysaccharide transport machinery is a frequent cause of mutational bile resistance. However, selection on lethal concentrations of bile also provides bile-resistant isolates that are not mutants. We propose that such isolates derive from rare cells whose physiological state permitted survival upon encountering bile. This view is supported by single cell analysis of gene expression using a microscope fluidic system: batch cultures of *Salmonella* contain cells that activate stress response genes in the absence of DOC. This phenomenon underscores the existence of phenotypic heterogeneity in clonal populations of bacteria and may illustrate the adaptive value of gene expression fluctuations.

## Author Summary

This study describes mechanisms employed by the bacterium *Salmonella enterica* to survive bile: adaptation, mutation, and non-mutational preadaptation. Adaptation is easily observed in the laboratory: when a *Salmonella* culture is grown in the presence of a sublethal concentration of the bile salt sodium deoxycholate (DOC), the minimal inhibitory concentration of DOC increases. Adaptation appears to be associated to multiple changes in gene expression induced by DOC. Mutational bile resistance is also a common phenomenon: plating on agar containing a lethal concentration of bile yields bile-resistant colonies. Fluctuation analysis indicates that such colonies derive from bile-resistant cells present in the previous culture. However, selection on lethal concentrations of bile also provides bile-resistant isolates that are not mutants. Non-mutational preadaptation, a non-canonical phenomenon *a priori*, suggests that batch cultures contain rare *Salmonella* cells whose physiological state permits survival upon encountering bile. The view that non-mutational preadaptation may be a consequence of phenotypic heterogeneity is supported by the observation that *Salmonella* cultures contain cells that activate stress response genes in the absence of DOC.

## Introduction

Bile is a fluid containing bile salts, cholesterol, and a variety of proteins and electrolytes [1]. Bile is synthesized by parenchymal cells (hepatocytes) in the liver. In mammals with a gall bladder, a fraction of bile is stored in the gall bladder while another fraction flows directly into the small intestine [1]. When food passes by the small intestine, gall bladder contraction releases bile into the duodenum. Bile aids in the digestion of fats, facilitates absorption of fat-soluble vitamins in the intestine, and contributes to the elimination of excess cholesterol and waste metabolic products produced in the liver [1].

About two thirds of bile (dry weight) are made of bile salts, a family of molecules with steroid structure which derive from cholesterol [2]. Bile salts dissolve membrane lipids and cause dissociation of integral membrane proteins. Inside the cell, the detergent activity of bile salts causes misfolding and denaturation of proteins [3], [4]. Chelation of calcium and iron by bile salts is also a source of physiological perturbations [3], [4]. Furthermore, bile salts have DNA damaging capacity, stimulate DNA rearrangements, and induce plasmid curing [4], [5], [6], [7]. However, certain bacterial species are resistant to the antibacterial activities of bile salts [3], [8]. This trait has been exploited for the design of selective microbiological media such as the one-century-old MacConkey agar used in the identification of genera of the family Enterobacteriaceae. On the other hand, bile salts regulate the expression of specific bacterial genes, some of them necessary for bile resistance and others involved in pathogenesis [3], [8]. Bile salts may thus be viewed both as environmental signals used by bacteria to identify bile-containing animal environments and as antibacterial compounds [8].

An extreme example of bile-resistant pathogen is *Salmonella enterica*, which colonizes the hepatobiliary tract during systemic infection and persists in the gall bladder during chronic infection [9], [10]. *Salmonella* survival in the mammalian gall bladder seems to involve several strategies. Invasion of the gall bladder epithelium may permit escape from the extremely high concentrations of bile salts present in the gall bladder lumen [11]. Formation of biofilms on gallstones may also protect *Salmonella* from the bactericidal activities of bile salts [12], [13]. However, planktonic *Salmonella* cells are also found at high numbers in the bile-laden gall bladder lumen, and the mechanisms employed to thrive in such a harsh environment remain to be identified.

Bile resistance can be studied under laboratory conditions by adding ox bile or individual bile salts to microbiological media [14]. Genetic and biochemical analysis in *E. coli* and *S. enterica* in the laboratory has permitted the identification of cell functions and mechanisms involved in bile resistance [3], [8], [14]. The relevance of these reductionist studies is supported by the fact that mutations that cause bile sensitivity *in vitro* often result in virulence attenuation in the mouse model of *S. enterica* infection [14]. The list of bile resistance factors in *Salmonella* and other enteric species includes envelope barriers such as the lipopolysaccharide [15], [16] and the enterobacterial common antigen [17], the outer membrane [18], [19], the cytoplasmic membrane [20], efflux pump systems [21], genes of the multiple antibiotic resistence (*mar*) and PhoPQ regulons [22], [23], and DNA repair functions [5], [6]. Genetic analysis has also identified cell functions whose loss increases bile resistance, probably by activating cell defense responses [20].

The bile resistance level of wild type *Salmonella* can be increased over the customary minimal inhibitory concentration by growth in the presence of sublethal concentrations of bile [8], [24]. This phenomenon, henceforth called “adaptation”, is easily observed in the laboratory and may be relevant during *Salmonella* colonization of the hepatobiliary tract. Below we describe studies of *Salmonella* adaptation to bile *in vitro*, and show that growth of *S. enterica* on sublethal concentrations of bile is accompanied by dramatic changes in gene expression. We also report that batch *Salmonella* cultures contain cells that show high levels of bile resistance without previous adaptation. This phenomenon, henceforth called “preadaptation”, seems to involve two unrelated processes. One is mutation in specific loci, often related to lipopolysaccharide transport; another is activation of bile resistance responses in a subpopulation of bacterial cells. The latter phenomenon fits well in current views indicating that bacterial populations are heterogeneous, and that fluctuations in gene expression can have adaptive value [25], [26].

## Results

### Viability of *S. enterica* SL1344 in the presence of sodium deoxycholate

**Figure 1:**
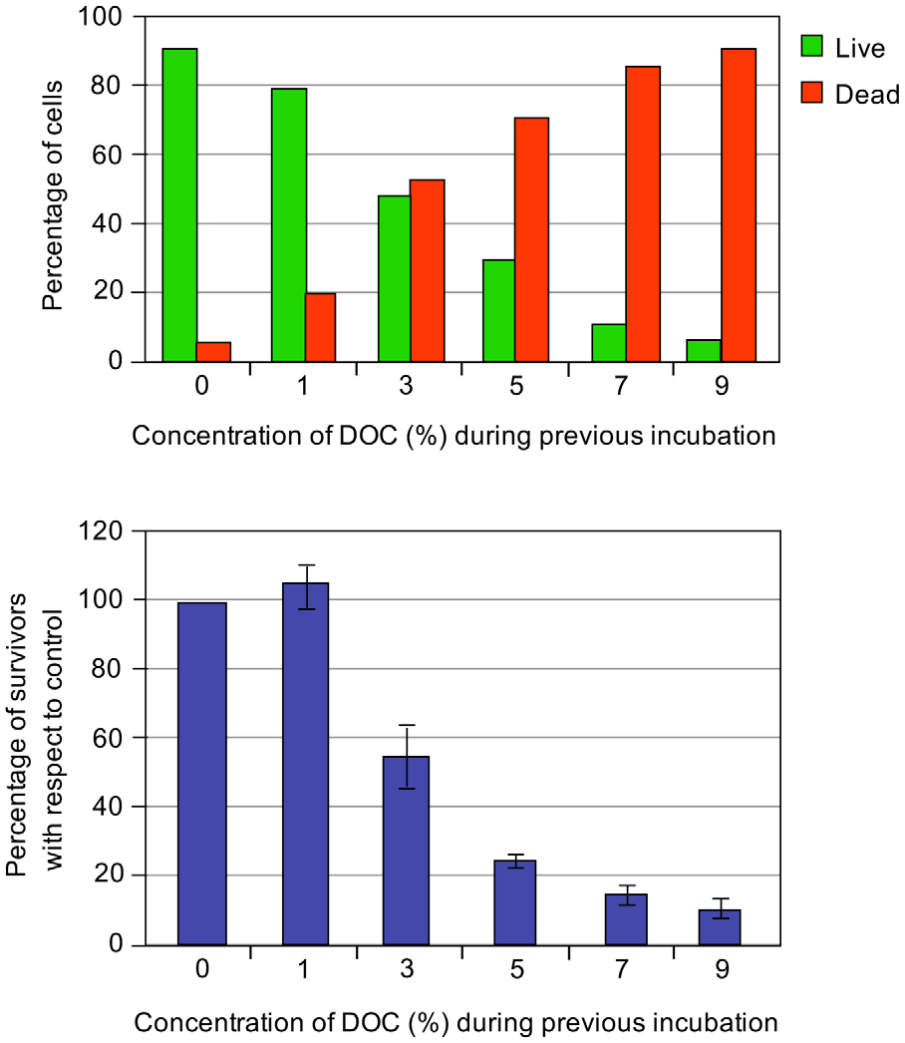
Percentages of live and dead bacteria and relative numbers of colony-forming units. Top panel: Percentages of live and dead bacteria (green and red histograms, respectively) found in 1 ml aliquots of an exponential culture of *S. enterica* SL1344 incubated in the presence of different concentrations of sodium deoxycholate (1%, 3%, 5%, 7% and 9%) during 30 minutes at 37°C. Bottom panel: Relative numbers of colony forming-units (CFU) after incubation of *S. enterica* SL1344 in the presence of different concentrations of sodium deoxycholate (1%, 3%, 5%, 7% and 9%) during 30 minutes at 37°C. The number of CFU in the absence of DOC is shown as 100%.

The minimal inhibitory concentration (MIC) of sodium deoxycholate for *S. enterica* strain SL1344 grown in LB is 7%, and the MIC of ox bile is 12% under the same conditions. These MICs are similar to those previously reported for strain ATCC 14028 [5], [6]. To ascertain whether inhibition of bacterial growth by bile salts involves bacterial death or merely growth arrest, we performed viability tests to distinguish live and dead *Salmonella* cells in the presence of DOC. Aliquots from *Salmonella* exponential cultures grown in LB were treated with various concentrations of DOC (1%, 3%, 5%, 7%, and 9%) for 30 min. Examination under the microscope using a commercial live/dead color-based kit was then performed, and the numbers of live/dead cells were counted. Cell counting was randomly performed, and the minimal number of bacterial cells counted was 1,700. A representative experiment is shown in Figure 1, top panel. A direct correlation was found between the percentage of dead *Salmonella* cells and the concentration of DOC.

The bactericidal capacity of bile was also monitored by plaque counts of colony-forming-units. Aliquots from *Salmonella* exponential cultures grown in LB were treated with various concentrations of DOC (1%, 3%, 5%, 7%, and 9%) for 30 min. The cultures were then diluted, plated on LB, and incubated overnight at 37°C. Colony counts confirmed that exposure to DOC renders *S. enterica* cells inviable in a dose-dependent maner (Figure 1, bottom panel).

### Adaptation of *S. enterica* to lethal concentrations of sodium deoxycholate

**Figure 2:**
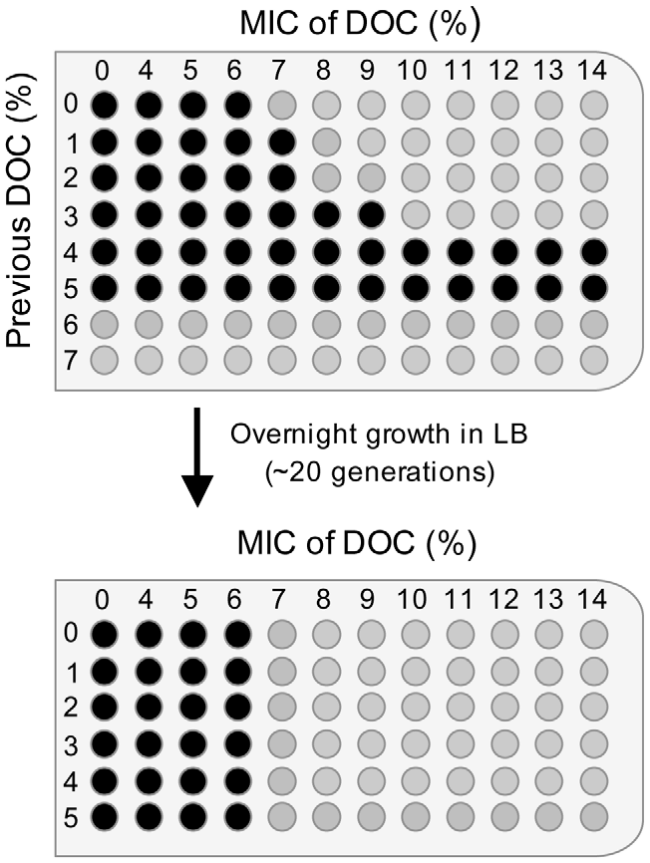
Minimal inhibitory concentrations (MICs) of sodium deoxycholate (DOC) for *Salmonella* cultures pre-exposed to various concentrations of DOC, and MICs for the same cultures after overnight growth in LB.

Although the level of resistance to bile is fairly constant under given conditions, *Salmonella* can be adapted to grow at higher concentrations of bile by previous growth in the presence of sublethal concentrations [8]. To determine the concentration(s) of DOC that permit adaptation in strain SL1344, *S. enterica* cultures were grown in LB containing different concentrations of DOC (from 1% to 7%). Aliquots from the cultures were then transferred to microtiter plates containing DOC at concentrations ranging from 1% to 14%. As shown in the diagram of Figure 2, growth at concentrations slightly lower than the MIC (4% and 5%) increased the MIC of DOC to ≥14%. A smaller increase of the MIC was likewise observed after growth in 3% DOC.

The bacterial cells that had survived bile in the microtiter plate were transferred to LB and cultured overnight. Aliquots from these cultures were then used to determine the MIC of DOC in microtiter plates. As shown in Figure 2, a MIC of 7% DOC was determined for all cultures, indicating that resistance had decreased back to the level characteristic of strain SL1344. Hence, adaptation to DOC by growth at concentrations of 3–5% is reversible, and does not involve selection of bile-resistant mutants, at least under the conditions tested.

### Transcriptomic analysis of *S. enterica* gene expression during growth on sublethal concentrations of sodium deoxycholate

The capacity of bile salts to induce changes in gene expression is well known in *S. enterica* [22], [27] and in other bacteria [3], as well as in eukaryotes [28]. On these grounds, we hypothesized that the reversible increase of MIC observed when *S. enterica* is grown in the presence of sublethal concentrations of DOC might involve changes in gene expression. This hypothesis was tested by transcriptomic analysis using the Salgenomics microarray [29].

RNA extraction was performed in exponential and stationary cultures grown in LB with and without 5% DOC (O.D._600_ = 0.4 and O.D._600_≥1, respectively). *S. enterica* grew at slower rates in LB+DOC than in LB, and the concentration of bacterial cells in LB+DOC reached a plateau well below the LB control. However, growth did occur, thus indicating that 5% is a sublethal concentration of DOC under such conditions (LB medium, 37°C). Note that these conditions are different from those used in the viability tests described above. This does not exclude, of course, that a fraction of bacterial cells may have been killed, and that the culture derives from the surviving subpopulation. Under these conditions, a large number of *Salmonella* loci showed differences in their RNA levels depending on whether DOC was present or absent. Raw data from transcriptomic analysis in the presence of 5% DOC have been deposited at the Array Express database (http://www.ebi.ac.uk/miamexpress) with accession number E-MTAB-637. Relevant data are summarized in Table 1 and Table 2. The main conclusions from these experiments can be summarized as follows:

- The RpoS-dependent genes *osmY*, *dps*, *uspB*, and *ecnB* [30] were found to be strongly upregulated by DOC in exponential cultures. The poorly characterized RpoS-dependent gene *ybiF*, located next to *dps* on the *Salmonella* chromosome, showed also >3 fold upregulation. In stationary cultures, *dps* and *osmY* were also upregulated by DOC. Their lower upregulation under such conditions is consistent with the fact that the RpoS-dependent general stress response is activated in non dividing cells [31]. These observations suggested that activation of the RpoS-dependent general stress response might play a role in adaptation to bile. A previous transcriptomic study in strain ATCC 14028 did not detect RpoS activation upon exposure to DOC [22]. However, the concentration of DOC used by Prouty et al. (3% ox bile) [22] may have been insufficient to trigger RpoS activation. In fact, under the conditions used in our study, concentrations of DOC much higher than those expected to be present in 3% ox bile (around 1% bile salts [1]) did not adapt *S. enterica* to bile (Figure 2).
- The general stress response is not the only stress response activated by sublethal concentrations of bile salts: the stress-inducible *cspD* gene [32], [33] was found to be upregulated by DOC in both exponential and stationary cultures, and the *uspA* gene [34] in stationary cultures only.
- The outer membrane protein (OMP) genes *ompC* and *ompD* [35] were downregulated by DOC during exponential growth. Because porins provide passage to bile salts [8], downregulation of *ompC* and *ompD* may be tentatively interpreted as a defensive modification of the outer membrane to decrease uptake of bile salts.
- Modification of the cell envelope may not be limited to outer membrane remodeling. Downregulation of *mltB*, a gene involved in peptidoglycan recycling [36], may provide evidence for cell wall changes produced in response to DOC. The crucial role of the cell envelope in bile resistance is well established in the literature [15], [17], [18], [19], [37].
- A sublethal concentration of DOC upregulated *acrD*, which encodes a component of a multidrug resistance efflux pump [38]. Because efflux systems are known to transport bile salts outside the cell [21], upregulation of *acrD* may be viewed as another defensive response. Upregulation of other transport genes (*ugpB* and *pnuC*) was also observed but it is difficult to interpret.
- *Salmonella* pathogenicity islands SPI-1 and SPI-2 were strongly downregulated by DOC, as previously described by other authors [27], [39]. Downregulation was observed in stationary cultures only, a result consistent with the fact that neither SPI-1 nor SPI-2 are expressed in exponential cultures [40].
- Miscellaneous gene expression changes of difficult interpretation are also presented in Table 1 and Table 2. Changes in the synthesis of cytochrome components may suggest alterations in electron transport. The possibility that chemotaxis is altered in the presence of DOC may be also considered, as previously proposed [22]. Altered expression of numerous metabolic genes is also observed.

**Table 1:** *S. enterica* loci showing altered expression (>3 fold) in the presence of 5% DOC during exponential growth.

Locus	Function of product	Fold change
*aroG*	Phenylalanine biosynthesis	+9.94
*osmY*	RpoS-dependent general stress response	+7.10
*dps*	RpoS-dependent general stress response	+7.04
*ecnB*	Entericidin synthesis	+6.28
*ugpB*	ABC transporter	+5.02
*yiiU*	Unkown function	+4.58
*cspD*	Stress response	+3.55
*cyoA*	Cytochrome oxidase	+3.39
*cfa*	Fatty acid synthesis	+3.22
*pnuC*	Nucleoside transport	+3.11
*acrD*	Efflux pump	+3.00
*sseA*	SPI-2 virulence effector	−12.01
*malZ*	Maltose catabolism	−5.28
*ssaS*	Component of the SPI-2 secretion apparatus	−4.48
*ompC*	Outer membrane	−3.42
*ompF*	Outer membrane	−3.39
*ibpA*	Heat shock	−3.02
*cheM-cheW*	Chemotaxis	−3.01
*mltB*	Peptidoglycan synthesis	−3.00

**Table 2:** *S. enterica* loci showing altered expression (>3 fold) in the presence of 5% DOC during stationary phase.

Locus	Function of product	Fold change
*cyoA*	Cytochrome oxidase	+26.33
*mdh*	Central metabolism	+12.02
*tsr*	Chemotaxis	+10.38
*shdB*	Central metabolism	+8.23
*sucA*	Central metabolism	+8.23
*ugpB*	ABC transporter	+7.40
*ompF*	Outer membrane	+7.34
*uspA*	Stress response	+6.50
*dps*	RpoS-dependent general stress response	+5.42
*filE-fliF*	Flagellum	+5.08
*narG-narK*	Nitrate reduction	+4.63
*ahpC-ahpF*	Stress response	+4.62
*nagB-nagE*	PTS system	+4.56
*uspB*	RpoS-dependent general stress response	+4.06
*cspD*	Stress response	+3.70
*osmY*	RpoS-dependent general stress response	+3.35
*nirD-nirC*	Nitrite reduction	+3.26
*phdR*	Central metabolism	+3.24
*ppa*	Central metabolism	+3.19
*fpb*	Central metabolism	+3.18
*rrmA*	Ribosomal RNA modification	+3.17
*cydA*	Cytochrome oxidase	+3.17
*pipA-pibB*	SPI-1 virulence effectors	−119.08
*sseA*	SPI-2 virulence effector	−68.34
*sopE*	SPI-1 virulence effector	−63.96
*sifB*	SPI-2 virulence effector	−54.29
*ssaS-ssaT*	SPI-2 secretion apparatus	−39.73
*ssaB*	SPI-2 secretion apparatus	−17.48
*hilC*	SPI-1 regulatory protein	−12.85
*prgH*	SPI-1 regulatory protein	−10.51
*hilD*	SPI-1 regulatory protein	−10.50
*pagC*	Intracellular survival in macrophages	−9.94
*iroN*	Siderophore	−9.23
*sseJ*	SPI-2 virulence effector	−7.91
*valW*	tRNA	−6.41
*potC*	Spermidine/putrescine transporter	−6.08
*sifA*	SPI-2 virulence effector	−6.08
*sifB*	SPI-2 virulence effector	−5.17
*invH*	SPI-1-encoded outer membrane lipoprotein	−5.10
*cheM-cheW*	Chemotaxis	−4.33
*hilA*	SPI-1 regulatory protein	−4.14
*tnpA*	IS*200* transposase	−4.09
*traX*	Conjugal transfer of the virulence plasmid	−3.39
*pagD*	Resistance to antimicrobial peptides	−3.18
*pyrI*	Central metabolism	−3.15

### Validation of microarray analysis using *lac* fusions

**Figure 3:**
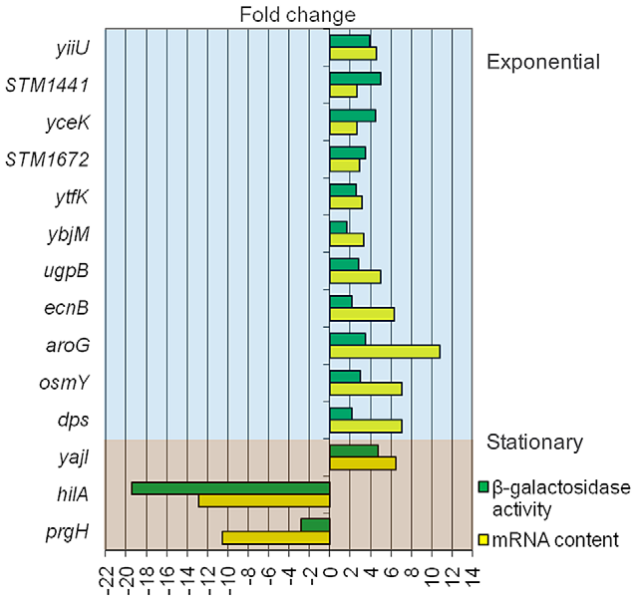
Validation of transcriptomic analysis: comparison of gene expression differences between LB and LB+5% deoxycholate as measured by RNA content (microarray analysis) and activity of *lac* fusions.

Data provided by transcriptomic analysis were validated by monitoring the effect of DOC on the expression of transcriptional *lac* fusions in 14 *S. enterica* genes. As a general rule, we chose genes which had shown strong expression changes in the presence of DOC. The sample included genes of the RpoS regulon (*osmY*, *dps*, and *ecnB*), a transport gene (*ugpB*), a metabolic gene (*aroG*), and two SPI-1 genes (*hilA* and *prgH*). Genes of unknown function, present in all enterics (*yiiU*, *yceK*, *ytfK*, *ybjM*, and *yajI*) or *Salmonella*-specific (*STM1441* and *STM1672*), were also included. The selection of these loci was based on the bile-sensitive phenotype of their mutants (e. g., *yiiU*), their DNA sequence relatedness to known bile resistance genes (e. g., *STM1441*, encoding a putative efflux pump) or the cellular location of their products (e. g., *yceK*, *yajI* and *STM1642*, which may encode outer membrane proteins, and *ybjM*, which may encode a cytoplasmic membrane protein). ß-galactosidase activities were measured in LB and in LB containing 5% DOC. Raw data are shown in Tables S1 and S2. Figure 3 is an elaboration that compares the ß-galactosidase activities of the *lac* fusions and the expression levels of the corresponding mRNAs detected by microarray analysis. Although differences in expression levels are observed, a correlation between mRNA content and ß-galactosidase activity is found in all cases.

Activation of the RpoS-dependent general stress response in the presence of sublethal concentrations of DOC was further analyzed by monitoring expression of RpoS-dependent genes other than *dps* and *osmY*. An additional goal of these experiments was to confirm that DOC upregulated the RpoS regulon in both exponential and stationary cultures. For these experiments, *lac* fusions in *katE*, *xthA*, *ots*, *dps*, and *osmY* were used. ß-galactosidase activities were measured in LB and in LB containing 5% DOC. Expression in exponential cultures was tested at an O.D._600_ = 0.4. Stationary cultures were tested at an O.D._600_≥1. Data shown in Table 3 and Table 4 confirmed that the RpoS regulon is upregulated by DOC. Upregulation occurs in both exponential and stationary cultures, and the induction ratios vary depending on the gene under study.

**Table 3:** ß-galactosidase activities of *lac* fusions in RpoS-regulated genes in the presence and in the absence of 5% sodium deoxycholate during exponential growth.

Strain	Gene fusion	LB	LB+DOC
SV6065	*katE::lac*	104±33	758±121
SV6888	*katE::lac rpoS*	10±3	18±3
SV6066	*ots::lac*	66±5	267±34
SV6067	*xthA::lac*	5±1	13±5
SV6068	*osmY::lac*	17±4	95±9
SV6069	*dps::lac*	5±2	12±2

ß-galactosidase activities are shown in Miller units. Data are averages and standard deviations from 3–5 independent experiments.

**Table 4:** ß-galactosidase activities of *lac* fusions in RpoS-regulated genes in the presence and in the absence of sodium deoxycholate in stationary phase.

Strain	Gene fusion	LB	LB+DOC
SV6065	*katE::lac*	512±88	1204±104
SV6888	*katE::lac rpoS*	14±6	22±5
SV6066	*ots::lac*	200±74	1575±220
SV6067	*xthA::lac*	32±4	63±11
SV6068	*osmY::lac*	105±27	370±49
SV6069	*dps::lac*	30±10	71±14

ß-galactosidase activities are shown in Miller units. Data are averages and standard deviations from 3–5 independent experiments.

### Identification of bile-responsive genes necessary for bile resistance

The identification of genes whose expression was altered in the presence of sublethal concentrations of DOC raised the possibility that such loci might be necessary for bile resistance. We thus tested the MIC of sodium deoxycholate for mutants carrying loss-of-function mutations in 16 genes identified above as upregulated by bile: *acrD*, *yiiU*, *STM1441*, *yceK*, *STM1672*, *ytfK*, *ybjM*, *ugpB*, *ecnB*, *aroG*, *osmY*, *dps*, *yajI*, *katE*, *ots*, and *xthA*. Results from these trials are shown in Table S3, and can be summarized as follows:

- The only bile-sensitive mutant was YiiU^−^ (MIC of DOC≅1.5%, compared with 7% in the wild type). Because this locus is virtually unknown, no explanation can be offered for its role in bile resistance.
- The observation that the AcrD^−^ mutant was not bile-sensitive is in agreement with previous observations made in strain ATCC 14028 [41], and can be explained by redundancy: *S. enterica* possesses multiple efflux systems, many of them versatile and with overlapping substrate specificity. Hence, bile sensitivity occurs only if multiple efflux systems are eliminated [41]. In agreement with this view, a TolC^−^ mutant (strain SV6629), which lacks an outer membrane protein of all RND efflux pumps [38], [41], showed extreme sensitivity to DOC (MIC≅0.02%, 350 fold lower than the MIC for the wild type). Extreme bile sensitivity of a TolC^−^ mutant has been likewise described in strain ATCC 14028 [41].
- Redundancy may also explain why mutants lacking individual RpoS-dependent genes (*osmY*, *dps*, *xthA*, *katE*, and *ots*) are not bile-sensitive. However, an RpoS^−^ derivative of SL1344 (strain SV5561) showed a MIC of DOC≅3%. Hence, an active RpoS regulon appears to be necessary for bile resistance but the individual RpoS-dependent functions tested in this study (OsmY, Dps, XthA, KatE, and Ots) are dispensable.

### Isolation of bile-resistant derivatives of *S. enterica* SL1344

Non adapted *Salmonella* populations (e. g., laboratory cultures in LB) are unable to grow on lethal concentrations of bile. However, bile resistance can be acquired by mutation, and bile-resistant mutants can be easily isolated upon plating on lethal concentrations of bile [14], [37]. However, previous descriptions of bile-resistant mutants had involved transposon insertions, which usually cause loss of function and are lethal if they occur in essential genes. To avoid these constraints, we isolated bile-resistant mutants of *S. enterica* of spontaneous origin. Aliquots from a *S. enterica* culture grown in LB were plated on LB supplemented with a lethal concentration of ox bile (180 g/l). Use of ox bile instead of DOC was justified by the fact that high concentrations of DOC prevent agar solidification. Bile-resistant colonies appeared at frequencies ranging between 10^−6^ and 10^−7^ per cell and generation. Because high concentrations of bile salts are bactericidal (Figure 1), we expected that bile-resistant colonies would derive from bile-resistant cells present in the previous culture. This hypothesis was supported by Luria-Delbrück fluctuation analysis [42]: the averages of bile-resistant colonies obtained from independent cultures showed a variance much higher than the averages from a single culture (Table S4).

**Figure 4:**
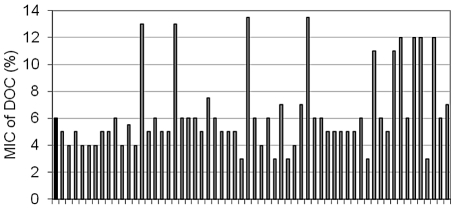
Minimal inhibitory concentration of sodium deoxycholate for bile resistant isolates after non-selective growth in LB. The isolates had been originally obtained on plates containing 18% ox bile.

Bile-resistant colonies were purified in LB and plated again on LB+bile to confirm bile resistance. During these routine purification procedures, we made the unexpected observation that a relatively large number of bile-resistant isolates had become bile-sensitive. A systematic analysis of the phenomenon was then carried out. Bile resistant isolates were obtained by plating strain SL1344 on LB+ox bile. Colonies were transferred to LB and grown overnight. The MIC of DOC for each isolate was then determined in microtiter plates. These trials confirmed that bile-resistant isolates were of two types: (i) stable, putatively carrying mutations that confer bile resistance; (ii) unstable isolates that lose bile resistance, either partially or completely, upon nonselective growth in LB. The frequencies of mutants and unstable isolates varied from one trial to another (data not shown). A representative experiment involving 59 independent bile-resistant isolates is shown in Figure 4. In this case, 10 isolates turned out to be mutants while the other 49 were unstable bile-resistant isolates.

### Characterization of bile-resistant mutant derivatives of *S. enterica* SL1344

Six spontaneous bile-resistant mutants (MIC of DOC ≥14%) of independent origin were chosen for full genome sequencing with the SOLiD platform [43]. Genome sequencing was followed by alignment with the *S. enterica* SL1344 genome sequence (ftp://ftp.sanger.ac.uk/pub/pathogens/Salmonella/STmSL1344.dbs) to identify DNA sequence differences. The genome of the laboratory stock of *S. enterica* SL1344 was also sequenced as a control. The mutations found are described in Table 5. Relevant observations are as follows:

- Four bile-resistant strains were single mutants. Mutants #1 and #6 harbored a nucleotide substitution and an in-frame deletion, respectively, in the *S. enterica yrbK* gene. YrbK (recently renamed LptC) is a lipopolysaccharide transport protein in *E. coli* [44]. Mutant #3 harbored a nucleotide substitution in the *rlpB* gene (also known as *lptE*), which encodes the *Salmonella* homolog of *E. coli* RlpB, a lipopolysaccharide assembly protein [45]. Mutant #4 harbored a nucleotide substitution in the poorly known *deaD* gene, which in *E. coli* encodes a putative ATP-dependent RNA helicase [46].
- Mutants #2 and #5 were double mutants. Interestingly, one of the mutations found in strain #2 mapped in the lipopolysaccharide transport gene *yrbK*. Strain #2 carried an additional mutation in a putative intergenic region of plasmid 2. In turn, mutant #5 carried frameshifts in two putative loci of unknown function (annotated as *strA* and *sul2*) on plasmid 3. These plasmids are specific of strain SL1344 and have not been described in other strains of serovar Typhimurium. A potential role of these plasmids in adaptation to bile seems unlikely, because strain ATCC 14028, which lacks the plasmids, shows MICs of DOC and bile virtually identical to those of SL1344 [5], [17], [19]. The occurrence of plasmid-borne mutations that cause bile resistance is thus interesting but difficult to interpret.

**Table 5:** Mutations present in the genomes of bile-resistant derivatives of *S. enterica* SL1344.

Mutant	Locus affected	Mutation^a^	Location of the mutation^b^	Predicted mutational change	Cellular function affected
1	*yrbK*	G→C substitution	Nucleotide 182	Arg→Pro	Lipopolysaccharide transport
2	*yrbK*	+1 frameshift	After nucleotide 399	Premature stop codon after amino acid 134	Lipopolysaccharide transport
2	Putative intergenic region on plasmid 2	A→G substitution	Base pair 13926	Unknown	Unknown
3	*rlpB*	C→A substitution	Nucleotide 287	Ala→Glu	Lipopolysaccharide transport
4	*deaD*	C→G substitution	Nucleotide 923	Ala→Gly	Putative ATP-dependent RNA helicase
5	*strA* locus on plasmid 3	+1 frameshift	Base pair 7949	Unknown	Unknown
5	*sul2* locus on plasmid 3	+1 frameshift	Base pair 38	Unknown	Unknown
6	*yrbK*	Deletion of 30 nucleotides	Base pairs 415–444	Loss of 10 amino acids	Lipopolysaccharide transport

a Nucleotide change is indicated for the coding sequence, when known.

b Base pair numbers are those of the annotated genome of *S. enterica* strain SL1344 (ftp://ftp.sanger.ac.uk/pub/pathogens/Salmonella/STmSL1344.dbs).

Bile-resistant mutants carrying mutations in known genes (*yrbK*, *rlpB*, and *deaD*) were reconstructed (see Materials and Methods for reconstruction procedures). All reconstructed mutants showed a MIC of DOC ≥14%, thus confirming that single mutations in *yrbK*, *rlpB*, and *deaD* caused the bile-resistant phenotype of these isolates. The cause of bile resistance in the DeaD^−^ mutant was not further investigated since *deaD* is a poorly known gene [46]. In contrast, the high frequency of mutations found in lipopolysaccharide transport genes (3 in *yrbK* and 1 in *rlpB*) provided evidence that alterations in lipopolysaccharide transport can cause bile resistance. LPS transport genes are known to be essential in *E. coli* [47], [48]. If such is also the case in *S. enterica*, the mutations detected must be leaky. Leakiness may seem normal for the G→C (*yrbK*) and C→A (*rlpB*) substitutions detected in mutants #1 and #3, and even for the *yrbK* in-frame deletion detected in mutant #6. The mutation detected in strain #2 (*yrbK*), however, is a frameshift, a mutation type that often causes loss of function. In fact, the frameshift consists of a C insertion, and results in the formation of a premature stop codon (TAA) eight nucleotides downstream. However, these changes map near the C-terminal region, suggesting that a truncated YrbK protein may be leaky. The view that the C-terminal region of YrbK is dispensable is further supported by the observation that the in-frame deletion found in mutant #6 maps in the same region (Table 5).

### Analysis of lipopolysaccharide in bile-resistant mutants

**Figure 5:**
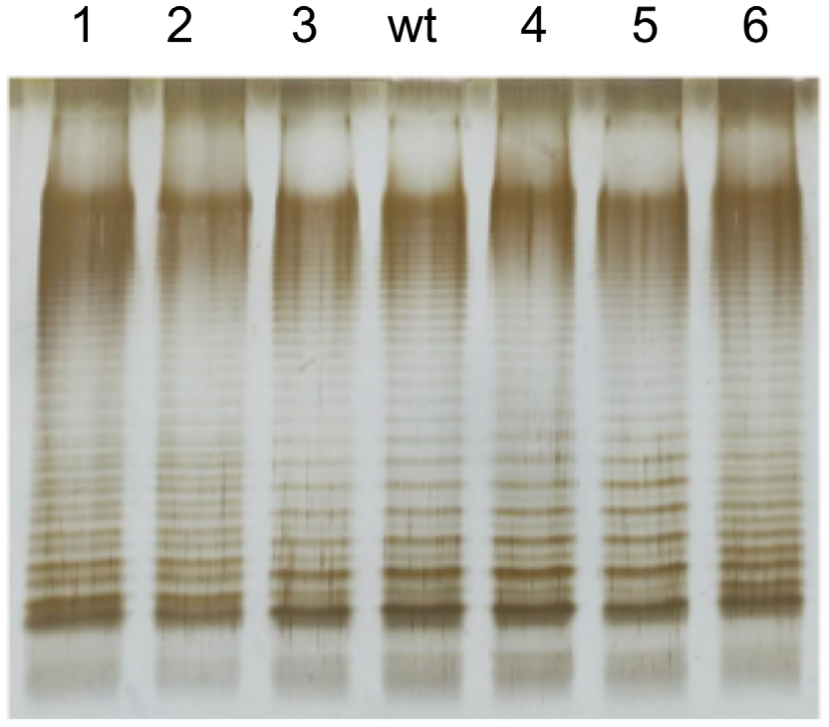
Lipopolysaccharide profiles of bile-resistant derivatives of *S. enterica* SL1344, as observed by electrophoresis and silver staining. The lane marked “wt” shows the LPS profile of the wild type strain. Lanes 1–6 show the LPS profiles of bile-resistant mutants #1, #2, #3, #4, #5, and #6.

Because the *rlpB* and *yrbK* genes have been described in *E. coli* as involved in LPS transport across the periplasm and LPS assembly at the outer membrane [49], we examined whether the bile-resistant mutants under study showed LPS alterations. The LPS of the DeaD^−^ mutant was also examined. Migration of the LPS in polyacrylamide gel is known to be affected by the number and size of repeating oligosaccharide units in long-chain LPS, such that bands in the profile represent progressively larger concatemers of the repeating oligosaccharide units [45]. Comparison of the LPS profiles of the mutants and the wild type shows that the *rlpB*, *yrbK, and deaD* mutations under study do not visibly alter the amount of LPS. However, structural differences are clearly observed between the wild type and mutants #1, #2, and #6, which carry *yrbK* mutant alleles (Figure 5). Reconstructed YrbK^−^ mutants showed LPS profiles identical to those of their parental mutants (Figure S1). The profiles found in these mutants may indicate differences in the oligosaccharide units that form long-chain LPS [48].

### Single cell analysis of gene expression in the presence and in the absence of sodium deoxycholate

**Figure 6:**
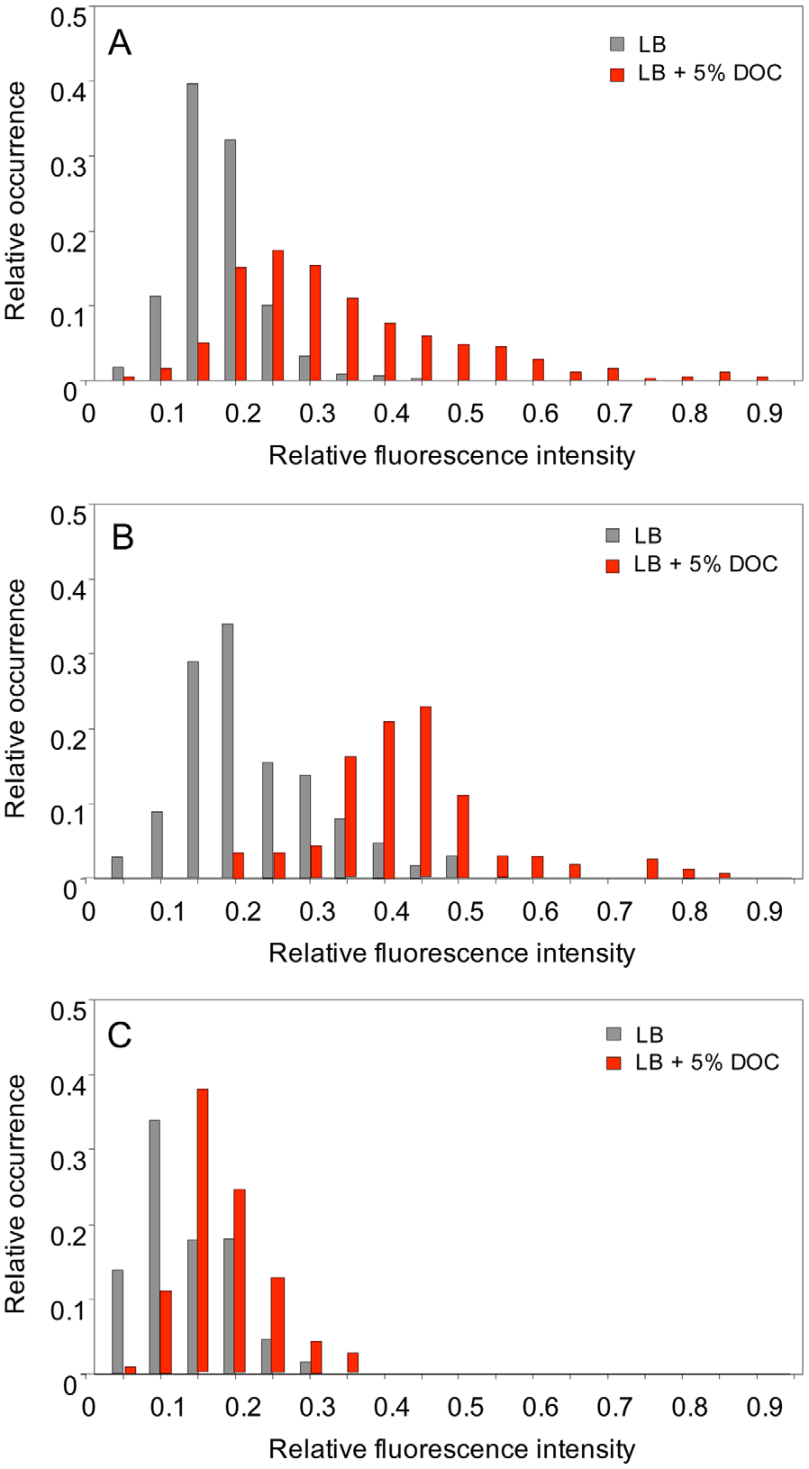
Levels of *osmY* gene expression in individual bacterial cells. Panels A and B show the distribution of fluorescence intensity in individual cells (*N*>300) of *S. enterica* SV6562 (*osmY*::GFP) in two independent experiments. In both cases, strain SV6562 was grown during 5 h in LB with or without 5% sodium deoxycholate. Histograms represent the proportion of bacterial cells showing distinct fluorescence levels in LB (grey) and LB+DOC (red). Fluorescence intensities are shown in an arbitrary scale (0–1). Panel C shows the distribution of fluorescence intensity in individual cells (*N*>300) of *S. enterica* SV6780 (*osmY*::GFP RpoS^−^) under conditions identical to those of experiments A and B.

**Figure 7:**
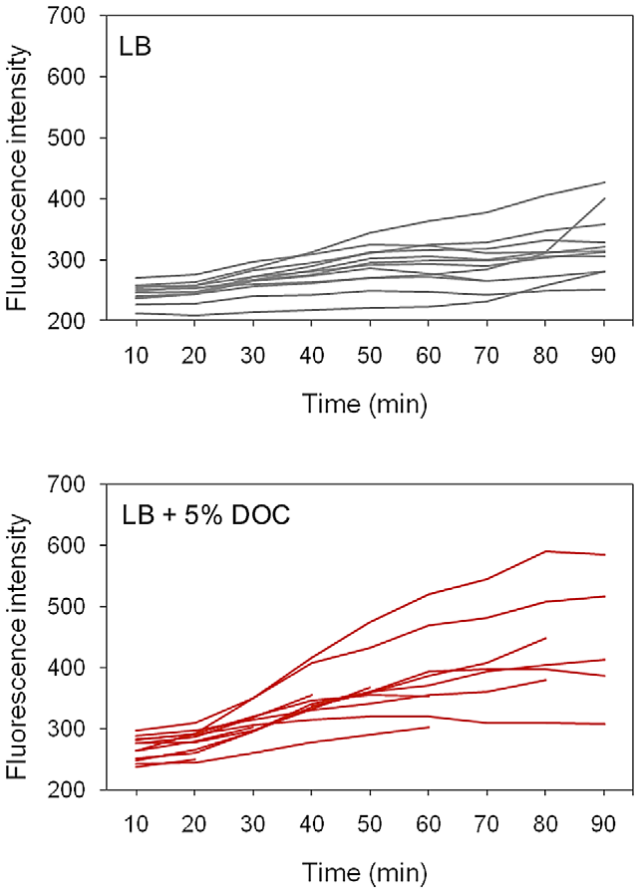
Time course of *osmY*::GFP expression in individual cells in the presence and in the absence of DOC. Aliquots from an exponential culture (O.D._600_ = 0.5) of *S. enterica* SV6562 (*osmY*::GFP) grown in LB were transferred to agar pads containing or not 5% sodium deoxycholate. Bacterial cells were fixed *in situ*, and GFP fluorescence intensity was measured at 10 min intervals during 90 min.

The observation that preadaptation of *S. enterica* to bile can occur by reversible, non mutational mechanisms raised the possibility that the bacterial population might contain cells which activate bile resistance responses in the absence of bile. This hypothesis was initially tested by examining the expression level of *osmY*, a gene of the RpoS regulon, in single *Salmonella* cells grown in the presence and in the absence of a sublethal concentration of DOC (5%, the same concentration used for transcriptomic analysis). To monitor *osmY* expression, a green fluorescent protein (GFP) fusion was constructed at the 3′ end of the *osmY* coding sequence (strain SV6562). Expression of the *osmY*::GFP fusion in individual *Salmonella* cells was monitored using a microscope automated fluidic system [50]. These experiments were of two kinds:

- Single point experiments: *S. enterica* cultures grown in LB and LB+5% DOC were diluted and transferred to agar pads. Ten fields, each containing ≥30 cells, were manually defined, and the fluorescence level of individual cells was measured [50]. Two representative, independent experiments are shown in Figure 6A and 6B. Exposure to 5% DOC increased *osmY*::GFP expression in an heterogeneous but consistent manner (red histograms). Cells grown in the absence of DOC (grey histograms) showed lower and more homogeneous levels of *osmY*::GFP expression. However, a significant degree of heterogeneity was observed, indicating that some cells activate the RpoS general stress response in the absence of DOC. Comparison of panels A and B reveals that the number of cells that activate the RpoS general response in the absence of DOC varies from one experiment to another, thus providing further evidence that phenotypic heterogeneity occurs in batch cultures. As a control, we analyzed *osmY*::GFP expression in an RpoS^−^ background (strain SV6780). As expected, the level of expression of *osmY* was significantly lower in the absence of RpoS, and *osmY*::GFP induction by DOC was very modest in the RpoS^−^ mutant (Figure 6C).
- Time lapse experiments: *S. enterica* cultures grown in LB were diluted, transferred to microscope slides, and covered with agar pads containing either LB or LB+5% DOC. The fluorescence of individual cells was then monitored during 90 minutes. A representative experiment involving 14 *Salmonella* cells is shown in Figure 7. The presence of DOC increased the level of fluorescence, albeit at different levels in different cells (perhaps reflecting differences in the initial level of *osmY*::GFP induction). Lysis of some cells was observed in the presence of DOC. In the absence of DOC, the fluorescence levels were lower. However, heterogeneous expression of *osmY*::GFP was observed in the absence of DOC, as in the single point experiments shown in Figure 6.

These experiments suggest that *S. enterica* cultures grown in LB contain cells with elevated expression of the RpoS-dependent general stress response. This observation offers a tentative explanation for non mutational preadaptation to bile: when an aliquot from an LB culture is plated on a lethal concentration of bile, sustainment and/or amplification of the general stress response in certain cells may permit the formation of bile-resistant colonies.

**Figure 8:**
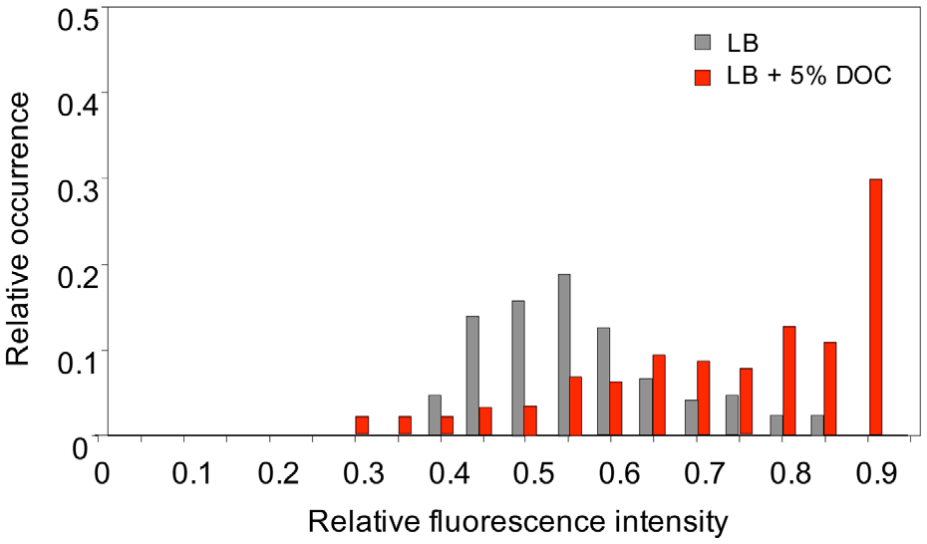
Levels of *cspD* gene expression in individual bacterial cells. The distribution of fluorescence intensity was measured in individual cells (*N*>300) of *S. enterica* SV6802 (*cspD*::GFP) after growth during 5 h in LB with or without 5% sodium deoxycholate. Histograms represent the proportion of bacterial cells showing distinct fluorescence levels in LB (grey) and LB+DOC (red). Fluorescence intensities are shown in an arbitrary scale (0–1).

To investigate whether heterogeneous gene expression occurred also in bile-responsive loci that do not belong to the RpoS regulon, we monitored expression of *cspD* [32], [33], a stress response gene which is upregulated by exposure to a sublethal concentration of DOC (Table 1 and Table 2). For this purpose, a GFP fusion was constructed at the 3′ end of the *cspD* coding sequence (strain SV6802). Single point experiments in LB and LB+5% DOC were carried out as above, and the fluorescence level of individual cells was measured [50]. Exposure to 5% DOC increased *cspD*::GFP expression in an heterogeneous but consistent manner (Figure 8). However, heterogeneity was also observed in LB (Figure 8), indicating that some cells activate *cspD* expression in the absence of DOC. Hence, phenotypic heterogeneity in gene expression is not restricted to RpoS regulon. This observation suggests that non mutational preadaptation to bile may require specific gene expression patterns, perhaps involving multiple loci.

## Discussion

Bacteria live in a changing environment, devoid of the homeostatic mechanisms that create stable conditions in the tissues of multicellular eukaryotes. Except for obligate parasites that have adapted to stable environments, survival of bacteria depends on ceaseless adaptation. This study describes mechanisms employed by *Salmonella enterica* to survive bile, a fluid with antibacterial capacity due to the presence of bile salts [3], [4], [8]. The adaptation mechanisms have been investigated using either ox bile, which contains a mixture of bile salts [1], or sodium deoxycholate, the most abundant and well known bile salt [2].

The concentrations of bile and DOC that inhibit *S. enterica* growth depend on the conditions used. For instance, it is well known that dividing cells are more sensitive to bile salts than non dividing cultures [19]. Furthermore, even under specific conditions, the minimal inhibitory concentration of bile increases if the *S. enterica* culture is previously grown on sublethal concentrations [8]. For instance, minimal inhibitory concentration analyses shown in Figure 2 indicate that growth on 4–5% DOC, a concentration which does not inhibit *Salmonella* growth, increases the MIC to ≥14%. Growth at DOC concentrations below 3% does not increase the MIC, and concentrations higher than 5% are inhibitory (Figure 2). Hence, sublethal (but relatively high) concentrations of DOC are necessary to increase the MIC above the standard inhibitory concentration.

A conceivable explanation for *Salmonella* adaptation to bile was that sublethal concentrations of bile might trigger changes of gene expression that could facilitate survival. Transcriptomic analysis in the presence of 5% DOC revealed indeed multiple changes in gene expression, some of which provided tentative explanations for the observed increase in bile resistance. Especially relevant was the observation that exposure to DOC activates the RpoS-dependent general stress response (Table 1, Table 2, Table 3, Table 4, and Figure 3). This response facilitates survival of *E. coli* and other gamma-proteobacteria under conditions that do not support active growth, and is also known to be activated by multiple stress conditions [31]. Hence it is not surprising that bile salts, which denature proteins and cause DNA damage [3], [4], can trigger the general stress response. The conclusion that the RpoS-dependent general stress response plays a crucial role in bile resistance is supported by two additional observations: (i) Lack of RpoS causes bile sensitivity (Table S3); (ii) *S. enterica* stationary cultures, in which the RpoS response is physiologically active, are more resistant to both DOC and ox bile [19]. The occurrence of extreme levels of resistance (≥14% DOC) in adapted cultures admits several explanations (not mutually exclusive). Activation of the RpoS regulon by sublethal concentrations of bile may be stronger than physiological activation in stationary cells. It is also possible that high levels of bile resistance result from simultaneous activation of more than one bile-resistance response. This latter possibility is supported by the observation that stress-inducible genes (*cspD, uspA, aphC*, etc.) that do not belong to the RpoS regulon are also activated by exposure to 5% DOC (Table 1 and Table 2). Furthermore, exposure to bile upregulates the expression of genes of unknown function, some of which might be part of stress response networks. An example is *yiiU*, which is activated by DOC (Table 1 and Figure 3) and is essential for bile resistance (Table S3).

High throughput analyses of gene expression also indicated that adaptation to bile may involve downregulation of porin genes and other genes encoding envelope structures, as well as upregulation of efflux pumps (Table 1 and Table 2). All these gene expression changes fit well in the literature: porins provide passage to bile salts [8], envelope structures are major barriers for bile salt uptake [8], and efflux pumps can transport bile salts outside the cell [21]. Hence, growth on sublethal concentrations of bile may permit *Salmonella* adaptation to lethal concentrations by triggering multiple changes in gene expression. Downregulation of pathogenicity island 1 by sublethal concentrations of DOC (Table 2), a phenomenon previously described [27], may be viewed as a signalling system used by *Salmonella* to identify environments that are not appropriate for epithelial cell invasion (e. g., the duodenum). In turn, downregulation of pathogenicity island 2 in the presence of bile (Table 2) may be viewed as a response that prevents activation of *Salmonella* functions involved in intracellular survival.

Preadaptation of *S. enterica* to bile is a completely different phenomenon, which does not pertain to *Salmonella* populations but to individual bacterial cells. Preadaptation is easily observed under laboratory conditions: when an aliquot of a *S. enterica* batch culture is plated on LB agar containing a lethal concentration of ox bile, bile-resistant colonies appear at frequencies ranging from 10^−6^ to 10^−7^ per cell and generation. These numbers fall in the known range of bacterial mutation frequencies [51]. Not surprisingly, Luria-Delbrück fluctuation analysis confirms that such colonies arise from bile-resistant cells found in the culture aliquot used for inoculation (Table S4). However, we were surprised to find that the majority of bile-resistant isolates obtained under such conditions were unstable, and lost bile resistance if grown overnight in LB without bile. Hence, preadaptive bile resistance seems to involve two distinct phenomena: mutation and non mutational preadaptation.

Full genome sequencing of 6 bile-resistant mutants revealed that 3 mutants carried mutations in *yrbK* and one in *rlpB*. The high frequency of mutations in lipopolysaccharide transport genes leaves little doubt that altered LPS transport can cause bile resistance. However, the identification of such mutants has intriguing aspects. One comes from the fact that LPS transport genes are known to be essential in *E. coli* [47], [48]. If such is also the case in *Salmonella*, the mutations found (two nucleotide substitutions, one in-frame deletion and one premature stop codon relatively close to the 3′ end of the coding sequence) must all be leaky. Another intriguing question is related to the fact that LPS is a major barrier against bile salts [15]. However, it is conceivable that transport of LPS components across the envelope might sensitize the cell to bile salts, thus explaining why altered transport may confer bile resistance. An alternative, speculative explanation is that the LPS transport proteins altered in bile-resistant mutants might be also involved in LPS assembly and/or modification, and that specific mutations might boost bile resistance. This view may be tentatively supported by the observation that some of the bile-resistant mutants described in this study have altered LPS profiles (Figure 5 and Figure S1).

Non mutational preadaptation to bile was at first sight an intriguing phenomenon. How can a *Salmonella* batch culture contain cells that are bile-resistant without previous adaptation by growth at sublethal concentrations? However, it is well known that bacterial cultures, albeit genetically clonal, can contain subpopulations of cells with distinct patterns of gene expression [25], either as a consequence of epigenetic control [52] or as the result of stochastic fluctuations in gene expression [25], [53]. We thus hypothesized that non mutational preadaptation might be caused by activation of bile-resistance responses in the absence of bile. This hypothesis was tested by analyzing expression of *osmY*, an RpoS-dependent gene, and *cspD*, a stress response gene that does not belong to the RpoS regulon [32], [33], in individual *Salmonella* cells grown in the presence and in the absence of a sublethal concentration of DOC. Microscopic microfluidics [50] confirmed that exposure to DOC activates *osmY* and *cspD* expression in most *Salmonella* cells (Figure 6 and Figure 7). However, upregulation of *osmY* and *cspD* expression was also observed in subpopulations of *Salmonella* cells in the absence of DOC (Figure 6, Figure 7, and Figure 8). Non mutational preadaptation may thus result from activation of the RpoS-dependent general stress response and/or other stress responses in a cell subpopulation. Stress response activation may either be triggered by a stress situation encountered by individual cells (e. g., increased concentrations of harmful metabolic products) or be accidental. Repression of specific loci, which has not been addressed in this study, may also contribute to preadaptation. It is possible that non mutational preadaptation to environmental challenges is a common phenomenon, and that the acquisition of bile resistance described in this study is merely one example among many others. The widespread occurrence of phenotypic polymorphism in clonal populations of bacteria [25], [52], [53] may support this possibility.

At this stage, it is impossible to ascertain whether the three distinct modes of *Salmonella* adaptation to bile described in this study may occur or not upon infection of animals. Current evidence obtained in the mouse model of typhoid fever indicates that *Salmonella* cells can escape the high concentrations of bile salts found in the gall bladder lumen by invading the gall bladder epithelium [11] and by forming biofilms on the surface of gallstones [12], [13]. However, planktonic *Salmonella* cells are also found in the gall bladder lumen, and little is known about the mechanisms that permit their survival and multiplication. Our model envisions that planktonic *Salmonella* cells may adapt to the gall bladder lumen by changing their gene expression pattern. Passage by the small intestine and the liver, which contain bile concentrations much lower than those found in the gall bladder [1], [2], might facilitate adaptation in an analogous manner as growth on sublethal concentrations of DOC in the laboratory. Phenotypic heterogeneity and subpopulation formation may additionally contribute to adaptation by activating bile resistance responses prior to colonization of the hepatobiliary tract. Lastly, appearance of bile-resistant mutants may provide an alternative mechanism for *Salmonella* adaptation to the gall bladder. Because bile salts are mutagenic [5], [6] and the dose of a mutagen is the product of its concentration by the time of exposure [54], mutational adaptation of *Salmonella* to bile might be speeded up by bile itself, especially during long term infection of the bile-laden gall bladder. This phenomenon might be relevant during persistent and chronic infections, as found for instance in human carriers of *Salmonella* Typhi [10].

## Materials and Methods

### Bacterial strains, plasmids, bacteriophages, media, and culture conditions

Strains of *Salmonella enterica* serovar Typhimurium (often abbreviated as *S. enterica*) used in this study (Table 1) derive from the mouse-virulent strain SL1344. Strains SV6065, SV6066, SV6067, SV6068, SV6069, SV6629, and SV6745 were constructed by transducing alleles from ATCC 14028 or LT2 to SL1344. Transduction was performed with phage P22 HT 105/1 *int201* ([55] and G. Roberts, unpublished data). The P22 HT transduction protocol was described elsewhere [56]. To obtain phage-free isolates, transductants were purified by streaking on green plates, prepared according to Chan et al. [57], except that methyl blue (Sigma Chemical Co., St. Louis, Missouri) substituted for aniline blue. Phage sensitivity was tested by cross-streaking with the clear-plaque mutant P22 H5. Luria-Bertani broth (LB) was used as standard rich medium and E medium [58] as minimal medium. Solid media contained agar at 1.5% final concentration. Cultures were grown at 37°C. Aeration of liquid cultures was obtained by shaking in an orbital incubator. Deoxycholic acid (sodium salt) and sodium choleate (ox bile extract) were both from Sigma. Antibiotics were used at the final concentrations described previously [5]. A strain list is provided as Table 6.

**Table 6:** Strain list.

Strain	Genotype
SV5561	*rpoS*::Ap^r^
SV6065	*katE*::Mu*d*K (Km^r^)
SV6066	*ots*::Mu*d*J (Km^r^)
SV6067	*xthA::lacZ*
SV6068	*osmY::lacZ*
SV6069	*dps::lacZ*
SV6088	*hilA::lacZ*
SV6090	*prgH::lacZ*
SV6109	*STM1441::lacZ*
SV6112	*ybjM::lacZ*
SV6115	*ecnB::lacZ*
SV6118	*STM1672::lacZ*
SV6124	*yajI::lacZ*
SV6127	*ugpB::lacZ*
SV6261	*aroG::lacZ*
SV6267	*ytfK::lacZ*
SV6270	*yiiU::lacZ*
SV6292	*yceK::lacZ*
SV6435	*rlpB* (287 C→A)
SV6562	*osmY*::GFP (Cm^r^)
SV6629	*tolC*::Cm^r^
SV6745	*acrD*:: Km^r^
SV6780	*osmY*::GFP (Cm^r^) *rpoS*::Ap^r^
SV6802	*cspD*::GFP (Cm^r^)
SV6880	*yrbK* (182 G→C) *yrbL*–Km^r^–*mtgA*
SV6883	*yrbK* (399+) *yrbL*–Km^r^–*mtgA*
SV6884	*yrbL*–Km^r^–*mtgA*
SV6888	*katE*::Mu*d*K *rpoS*::Ap^r^
SV6889	*deaD* (923 G→C)

### Gene disruption and directed construction of *lac* fusions

Targeted gene disruption was achieved using plasmids pKD3, pKD4, and pKD13 [59]. Antibiotic resistance cassettes introduced during strain construction were excised by recombination with plasmid pCP20 [59]. The oligonucleotides used for disruption (labeled “FOR” and “REV”) are listed in Table S5 together with the oligonucleotides (labeled “E”) used for allele verification by the polymerase chain reaction. For the construction of transcriptional and translational *lac* fusions in the *Salmonella* chromosome, FRT sites generated by excision of Km^r^ cassettes were used to integrate either plasmid pCE36 or pCE40 [60].

### Construction of *osmY*::GFP and *cspD*::GFP fusions

An *osmY::gfp* fusion was constructed as described by Hautefort et al. [61]. A fragment containing the promoterless green fluorescent protein (*gfp*) gene and the chloramphenicol resistance cassette was amplified from the pZEP07 plasmid with primers 5′ AAG CCG TTG ATG GCG TAA AAA GTG TTA AAA ACG ATC TGA AAG TTC AGT AAT AAG AAG GAG ATA TAC ATA TGA G 3′, and 5′ GGT GCA CAT TAC GCC TCC CGA CAA ACG TCG GGA GGA CGA ATT ACG ACG AAT TAT CAC TTA TTC AGG CGT A 3′. Primers used for *cspD* amplification were 5′ GCA ATC ACG CCA GCG TCA TCG TGC CCA TCG AAG CAG AGG CCG TTG CAT AGT AAG AAG GAG ATA TAC ATA TGA G 3′, and 5′ CGA TCG GGC TGG CAT TTT GCC TCC TGG ATG TAC ACA ATG AGA CAG AGG AGT TAT CAC TTA TTC AGG CGT A 3′. The 5′ regions of these primers are homologous to the 3′ end of the *osmY* and *cspD* coding sequences, so that the fusion is formed immediately after the *osmY* and *cspD* stop codons. The constructs were integrated into the chromosome of *S. enterica* using the Lambda Red recombination system [59].

### Directed construction of *rlpB* and *deaD* point mutations

The *rlpB* allele from mutant #3 and the *deaD* allele from mutant #4 were PCR-amplified using pairs of 30-nucleotide primers that contained XbaI and SacI targets. The primers for *rlpB* amplification were 5′ TTT TGA GCT CGA AGG TGA TAT CGA CAA CGC 3′, and 5′ TTT TTC TAG ACT CAT TCA TTG CCG CGT TAG 3′; for *deaD* amplification, 5′ TTT TGA GCT CCG TCT GCT TGA TCA CTT AAA 3′, and 5′ TTT TTC TAG AAC GAC GTT CAC GAC GCG GAC 3′. The resulting fragments were digested with XbaI and SacI, cloned onto pDMS197 [61] and propagated in *E. coli* CC118 lambda *pir* [62]. Plasmids derived from pMDS197 were transformed into *E. coli* S17-1 lambda *pir* [63]. The resulting strains were used as donors in matings with *S. enterica* SL1344 as recipients, selecting Tc^r^ transconjugants on E plates supplemented with tetracycline and histidine. Several Tc^r^ transconjugants were grown in nutrient broth (without NaCl) containing 5% sucrose. Individual tetracycline-sensitive segregants were then examined for the incorporation of the mutant allele by DNA sequencing.

### Reconstruction of bile-resistant mutants by P22-mediated transduction of a linked marker

The kanamycin-resistant cassette of plasmid pKD4 was inserted at a region close to the mutation under study, using lambda Red recombination [59]. The oligonucleotides employed for gene targeting are listed in Table S5. To reconstruct *yrbK* mutations, the Km^r^ cassette was inserted at an intergenic region between *yrbL* and *mtgA*, 37 bp downstream the *yrbL* stop codon and 28 bp downstream the *mtgA* stop codon (note that *yrbL* and *mtgA* undergo divergent transcription). The distance from the Km^r^ cassette and the *yrbK* mutations under study is 6,135 bp for mutant #1, and 5,918 bp for mutant #2. To reconstruct the *rlpB* mutation of mutant #3, the Km^r^ cassette was introduced at an intergenic region between the putative ORFs *ybeL* and *ybeQ* (21 bp downstream the putative *ybeL* stop codon, and 8,253 bp away from the *rlpB* mutation). DNA sequence analysis employed the *S. Typhimurium* SL1344 genome database (ftp://ftp.sanger.ac.uk/pub/pathogens/Salmonella/STmSL1344.dbs).

The presence of the original mutation in the reconstructed mutants was verified by PCR amplification using primers designed *ad hoc* (Table S5), followed by DNA sequencing. In all constructions, the distance between the Km^r^ cassette and the mutation under study permitted >90% cotransduction of the Km^r^ cassette and the point mutation under study, fulfilling calculations made with the formula of Wu [62].

### Determination of minimal inhibitory concentrations of sodium deoxycholate

Exponential cultures in LB broth were prepared, and samples containing around 3×10^2^ colony-forming-units (CFU) were transferred to polypropylene microtiter plates (Soria Genlab, Valdemoro, Spain) containing known amounts of sodium deoxycholate (DOC). After 12 h incubation at 37°C, growth was visually monitored. Assays were carried out in triplicate. Student's *t*-test was used to analyze every MIC. The null hypothesis was that MICs were not significantly different from the MIC for the wild-type. *P* values of 0.01 or less were considered significant.

### Assessment of bacterial viability using a cell staining kit

One ml aliquots of an exponential culture of *S. enterica* SL1344 grown in LB were incubated in the presence of different concentrations of sodium deoxycholate (1%, 3%, 5%, 7% and 9%) during 30 minutes at 37°C. The cells were then washed three times with 0.85% NaCl and stained using the Viability/Cytotoxicity Assay Kit for Bacteria (Biotium Inc., Hayward, California). Control suspensions of live and dead cells were prepared as described in the kit protocol. Live and dead cells were distinguished using a Leica DMR 020-525.024 fluorescence microscope (Leica Camera AG, Solms, Germany). Live and dead bacteria were counted as the green and red cells (respectively) found in randomly selected 5×5 mm squares painted on a micro cover glass.

### Assessment of bacterial viability by plate counts

Aliquots of *Salmonella* exponential cultures grown in LB, each containing 2×10^6^ cells, were treated with various concentrations of DOC (1%, 3%, 5%, 7%, and 9%) for 30 min. The cultures were then diluted, plated on LB and incubated at 37°C. Counts of colony-forming-units were performed after overnight growth.

### Isolation of bile-resistant mutants and Luria-Delbrück fluctuation assays

Bile-resistant derivatives of *S. enterica* SL1344 were isolated by plating 0.1 ml aliquots (approximately, 2×10^8^ cells) from an overnight LB culture onto LB plates containing 18% ox bile (Sigma-Aldrich, St. Louis, Missouri). Fluctuation analysis was performed as described by Luria and Delbrück [42], and the number of independent cultures was 40.

### DNA isolation and full-genome sequencing

Whole genome DNA samples from bile-resistant mutants and from the parent strain SL1344 were prepared by phenol extraction and ethanol precipitation. Whole genome sequencing was performed using the oligonucleotide ligation and detection (SOLiD, v2) platform [43] at the facilities of Sistemas Genómicos S.L., Parque Tecnológico de Valencia, Paterna, Spain, using mate-pair libraries and reads of 25 nucleotides [63]. DNA sequences were aligned with the genome sequence of *Salmonella* Typhimurium SL1344 available at the Wellcome Trust Sanger Institute, Hinxton, England (ftp://ftp.sanger.ac.uk/pub/pathogens/Salmonella/STmSL1344.dbs). Two mismatches or mispairs per reading were permitted.

### RNA isolation, microarray procedures, and data analysis

To prepare cells for RNA extraction, 25 ml of fresh LB and LB+DOC 5% in a 250 ml flask was inoculated with a 1∶100 dilution from an overnight bacterial culture, and incubated with shaking at 250 rpm in a New Brunswick Innova 3100 waterbath at 37°C. Three biological replicates were performed for each strain, and RNA was extracted at an optical density (OD_600_)∼0.4 (exponential phase) and >1 (stationary phase). RNA extractions were performed as described by Mangan et al. [64], and their quality was assessed on an Agilent 2100 Bioanalyzer. Transcriptomic analyses were performed with the Salgenomics microarray [29]. Hybridization and microarray scanning were performed at the Genomics Service of the Centro Nacional de Biotecnología, C.S.I.C., Cantoblanco, Madrid, Spain (http://www.cnb.uam.es/content/services/genomics). For normalization of the two-color microarray data, LiMMA software [65] was used. Further bioinformatic analysis was carried out with the FIESTA programme (http://bioinfogp.cnb.csic.es/tools/FIESTA/index.php). Raw transcriptomic data were deposited at the Array Express database (http://www.ebi.ac.uk/miamexpress) under accession number E-MTAB-637.

### ß-galactosidase assays

Levels of ß-galactosidase activity were assayed as described by Miller [66], using the CHCl_3_-sodium dodecyl sulfate permeabilization procedure.

### Electrophoretic visualization of lipopolysaccharide profiles

To investigate lipopolysaccharide (LPS) profiles, bacterial cultures were grown in LB. Bacterial cells were harvested and washed three times with 0.9% NaCl. The O.D._600_ of the washed bacterial suspension was measured to calculate cell concentration. A bacterial mass containing about 3.2×10^8^ cells was pelleted by centrifugation. Treatments applied to the bacterial pellet, electrophoresis of crude bacterial extracts, and silver staining procedures were performed as described by Buendía-Clavería *et al.* [67]. Three replicates per strain were performed.

### Microscopy

Cells were inoculated onto a microscope cover slip and covered with a thin (2 mm thick) semisolid LB agar (1.5%) matrix with or without DOC. In the DOC-containing samples, the final concentration of DOC was 5%. The cover slip and the agar pad were then mounted in a seal flow chamber allowing constant aeration and reduced desiccation. Flow chambers were incubated in a temperature-controlled automated microscope (Nikon TE2000-E-PFS, Nikon, Champigny-sur-Marne, France) at 37°C. For single point experiments, 10 fields, each containing at least 30 cells treated or not with DOC, were manually defined. For 90 min time lapse experiments, a single field was examined, and time points were taken every 10 min under agar pads with or without DOC. Images were recorded using a CoolSNAP HQ2 high resolution camera (Roper Scientific, Evry, France) and a 100x/1.4 DLL objective. Digital analysis and image treatment were performed with Metamorph software 7.5 (Molecular Devices, Sunnyvale, California) as previously described [50].

## Supporting Information

Figure S1 LPS profiles of bile resistant mutants #1 and #2 (both carrying *yrbK* mutations), reconstructed by P22-mediated transduction of a linked Km^r^ marker. Lanes are as follows: 1, YrbK^+^ Km^r^ transductant obtained with a P22 HT lysate grown on SV6880 (*yrbK* G→C Km^r^); 2, YrbK^−^ Km^r^ transductant obtained with a P22 HT lysate grown on SV6880; wt, wild type; 3, YrbK^−^ Km^r^ transductant obtained with a P22 HT lysate grown on SV6883 (*yrbK* +1 frameshift Km^r^); YrbK^+^ Km^r^ transductant obtained with a P22 HT lysate grown on SV6883. Transductants 1 and 4 were bile-sensitive, while transductants 2 and 3 were bile-resistant. The *yrbK* mutations carried by transductants 2 and 3 were confirmed by PCR amplification and DNA sequencing of the amplified fragments.
(PDF)

Table S1 ß-galactosidase activities of *lac* fusions in bile-responsive genes during exponential growth in the presence and in the absence of DOC.
(DOC)

Table S2 ß-galactosidase activities of *lac* fusions in bile-responsive genes in stationary cultures grown in the presence and in the absence of DOC.
(DOC)

Table S3 Minimal inhibitory concentrations (g/100 ml) of sodium deoxycholate for *S. enterica* strains mentioned in this study, all derived from SL1344.
(DOC)

Table S4 Fluctuation in the frequencies of bile-resistant mutants obtained upon plating of *S. enterica* SL1344 on LB+18% ox bile.
(DOC)

Table S5 Oligonucleotides.
(DOC)
