# Supplementary figures and images for: Adaptation and Preadaptation of Salmonella enterica to Bile
Source: PLoS Genet. 2012 Jan 19;8(1):e1002459. doi: 10.1371/journal.pgen.1002459 (PMC3261920; doi:10.1371/journal.pgen.1002459)

1 2 wt 3 4

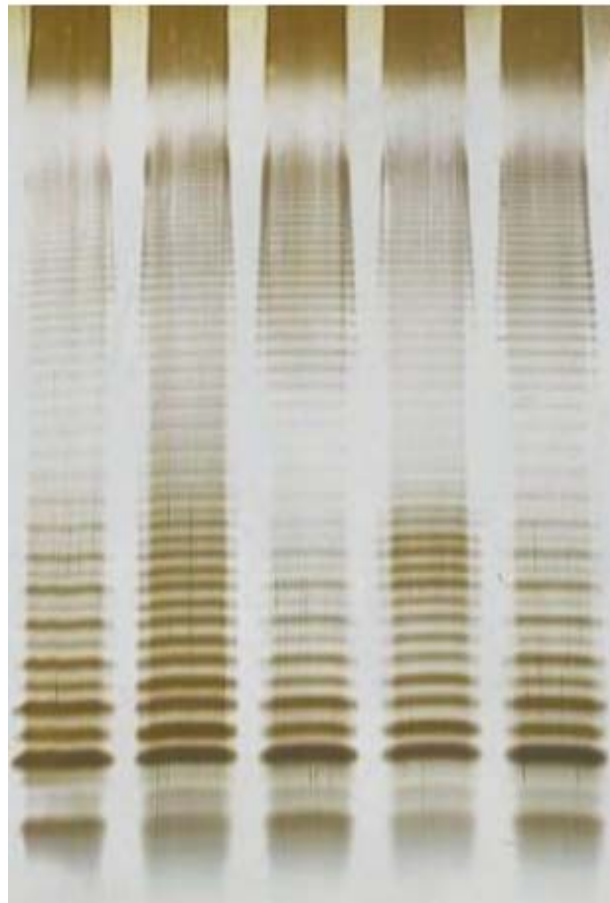

Supplement: Figure S1 — LPS profiles of bile resistant mutants #1 and #2 (both carrying yrbK mutations), reconstructed by P22-mediated transduction of a linked Kmr marker. Lanes are as follows: 1, YrbK+ Kmr transductant obtained with a P22 HT lysate grown on SV6880 (yrbK G→C Kmr); 2, YrbK− Kmr transductant obtained with a P22 HT lysate grown on SV6880; wt, wild type; 3, YrbK− Kmr transductant obtained with a P22 HT lysate grown on SV6883 (yrbK +1 frameshift Kmr); YrbK+ Kmr transductant obtained with a P22 HT lysate grown on SV6883. Transductants 1 and 4 were bile-sensitive, while transductants 2 and 3 were bile-resistant. The yrbK mutations carried by transductants 2 and 3 were confirmed by PCR amplification and DNA sequencing of the amplified fragments. (PDF) [file pgen.1002459.s001.pdf]
