# Supplementary material for: Adaptation and Preadaptation of Salmonella enterica to Bile
Source: PLoS Genet. 2012 Jan 19;8(1):e1002459. doi: 10.1371/journal.pgen.1002459 (PMC3261920; doi:10.1371/journal.pgen.1002459)
Supplement: Table S1 — ß-galactosidase activities of lac fusions in bile-responsive genes during exponential growth in the presence and in the absence of DOC. (DOC) [file pgen.1002459.s002.doc]

**Table S1.** ß-galactosidase activities of *lac* fusions in bile-responsive genes in exponential cultures grown in the presence and in the absence of sodium deoxycholate (DOC)

| Strain | Gene fusion | LB | LB + DOC |
| --- | --- | --- | --- |
| SV6068 | *osmY::lacZ* | 19± 0.7 | 80 ± 24 |
| SV6069 | *dps::lacZ* | 5 ± 0.2 | 11.5 ± 1.2 |
| SV6088 | *hilA::lacZ* | 38 ± 3 | 14 ± 2.5 |
| SV6090 | *prgH::lacZ* | 555 ± 60 | 208 ± 48 |
| SV6109 | *STM1441::lacZ* | 2.8 ± 0.1 | 14.8 ± 3.1 |
| SV6112 | *ybjM::lacZ* | 9.5 ± 3.5 | 15 ± 1.4 |
| SV6115 | *ecnB::lacZ* | 2480 ± 143 | 6700 ± 950 |
| SV6118 | *STM1672::lacZ* | 370 ± 14 | 1400 ± 124 |
| SV6124 | *yajI::lacZ* | 1.4 ± 0.07 | 6.2 ± 0.4 |
| SV6127 | *ugpB::lacZ* | 2.3 ± 05 | 4.8 ± 1 |
| SV6261 | *aroG::lacZ* | 97 ± 6 | 330 ± 100 |
| SV6267 | *ytfK::lacZ* | 360 ± 80 | 650 ± 140 |
| SV6270 | *yiiU::lacZ* | 1332 ± 66 | 7676 ± 835 |
| SV6292 | *yceK::lacZ* | 11 ± 0.2 | 45 ± 10 |

Cultures were prepared in LB and LB containing 5% DOC. Aliquots were extracted at O.D._600_ = 0.4. ß-galactosidase activities are shown in Miller units. Data are averages and standard deviations from 3 experiments.
