# Supplementary material for: Adaptation and Preadaptation of Salmonella enterica to Bile
Source: PLoS Genet. 2012 Jan 19;8(1):e1002459. doi: 10.1371/journal.pgen.1002459 (PMC3261920; doi:10.1371/journal.pgen.1002459)
Supplement: Table S2 — ß-galactosidase activities of lac fusions in bile-responsive genes in stationary cultures grown in the presence and in the absence of DOC. (DOC) [file pgen.1002459.s003.doc]

**Table S2.** ß-galactosidase activities of *lac* fusions in bile-responsive genes in stationary cultures grown in the presence and in the absence of sodium deoxycholate (DOC)

| Strain | Gene fusion | LB | LB + DOC |
| --- | --- | --- | --- |
| SV6068 | *osmY::lacZ* | 136 ± 12 | 344 ± 35 |
| SV6069 | *dps::lacZ* | 35.6 ± 0.36 | 64 ± 1.4 |
| SV6088 | *hilA::lacZ* | 330 ± 24 | 17 ± 1.4 |
| SV6090 | *prgH::lacZ* | 1026 ± 50 | 360 ± 28 |
| SV6109 | *STM1441::lacZ* | 0.9 ± 0.2 | 6.6 ± 2.6 |
| SV6112 | *ybjM::lacZ* | 22.3 ± 1.2 | 34.4 ± 2.8 |
| SV6115 | *ecnB::lacZ* | 10095 ± 504 | 12140 ± 689 |
| SV6118 | *STM1672::lacZ* | 1280 ± 42 | 1440 ± 130 |
| SV6124 | *yajI::lacZ* | 1.8 ± 0.5 | 8 ± 2 |
| SV6127 | *ugpB::lacZ* | 5.3 ± 0.7 | 6.5 ± 1.8 |
| SV6261 | *aroG::lacZ* | 46 ± 3 | 282 ± 25 |
| SV6267 | *ytfK::lacZ* | 960 ± 20 | 1020 ± 192 |
| SV6270 | *yiiU::lacZ* | 1278 ± 36 | 3957 ± 226 |
| SV6292 | *yceK::lacZ* | 7.2 ± 0.1 | 48 ± 4.2 |

Cultures were prepared in LB and LB containing 5% DOC. Aliquots were extracted at O.D._600_ ≥ 1. ß-galactosidase activities are given in Miller units. Data are averages and standard deviations from 3 experiments.
