# Supplementary material for: Adaptation and Preadaptation of Salmonella enterica to Bile
Source: PLoS Genet. 2012 Jan 19;8(1):e1002459. doi: 10.1371/journal.pgen.1002459 (PMC3261920; doi:10.1371/journal.pgen.1002459)
Supplement: Table S3 — Minimal inhibitory concentrations (g/100 ml) of sodium deoxycholate for S. enterica strains mentioned in this study, all derived from SL1344. (DOC) [file pgen.1002459.s004.doc]

**Table S3.** Minimal inhibitory concentrations (g/100 ml) of sodium deoxycholate for *S. enterica* strains mentioned in this study

| Strain | Genotype | M.I.C. |
| --- | --- | --- |
| SV5561 | *rpoS*::Ap^r^ | 3 |
| SV6065 | *katE*::Mu*d*K (Km^r^) | 7 |
| SV6066 | *ots*::Mu*d*J (Km^r^) | 7 |
| SV6067 | *xthA::lacZ* | 7 |
| SV6068 | *osmY::lacZ* | 7 |
| SV6069 | *dps::lacZ* | 7 |
| SV6109 | *STM1441::lacZ* | 7 |
| SV6112 | *ybjM::lacZ* | 7 |
| SV6115 | *ecnB::lacZ* | 7 |
| SV6118 | *STM1672::lacZ* | 7 |
| SV6124 | *yajI::lacZ* | 7 |
| SV6127 | *ugpB::lacZ* | 7 |
| SV6261 | *aroG::lacZ* | 7 |
| SV6267 | *ytfK::lacZ* | 7 |
| SV6270 | *yiiU::lacZ* | 1.5 |
| SV6292 | *yceK::lacZ* | 7 |
| SV6435 | *rlpB* (287 C → A) | >12 |
| SV6562 | *osmY*::GFP (Cm^r^) | 7 |
| SV6629 | *tolC*::Cm^r^ | 0.02 |
| SV6745 | *acrD*:: Km^r^ | 7 |
| SV6780 | *osmY*::GFP (Cm^r^) *rpoS*::Ap^r^ | 7 |
| SV6880 | *yrbK* (182 G → C) *yrbL*–Km^r^–*mgtA* | >12 |
| SV6802 | *cspD*::GFP (Cm^r^) | 7 |
| SV6883 | *yrbK* (399+) *yrbL*–Km^r^–*mgtA* | >12 |
| SV6884 | *yrbL*–Km^r^–*mgtA* | 7 |
| SV6888 | *katE*::Mu*d*K *rpoS*::Ap^r^ | 3 |
| SV6889 | *deaD* (923 G → C) | >12 |
