# Supplementary material for: Adaptation and Preadaptation of Salmonella enterica to Bile
Source: PLoS Genet. 2012 Jan 19;8(1):e1002459. doi: 10.1371/journal.pgen.1002459 (PMC3261920; doi:10.1371/journal.pgen.1002459)
Supplement: Table S4 — Fluctuation in the frequencies of bile-resistant mutants obtained upon plating of S. enterica SL1344 on LB+18% ox bile. (DOC) [file pgen.1002459.s005.doc]

**Table S4.** Fluctuation in the frequencies of bile-resistant colonies obtained upon plating of *S. enterica* SL1344 on LB + 18% ox bile

| Number of colonies | | |
| --- | --- | --- |
| Independent cultures | | Single culture |
| 12 | | 6 |
| 5 | | 9 |
| 6 | | 8 |
| 18 | | 7 |
| 1 | | 4 |
| 25 | | 1 |
| 310 | | 1 |
| 32 | | 22 |
| 2 | | 10 |
| 7 | | 12 |
| 1 | | 10 |
| 3 | | 10 |
| 4 | | 8 |
| 5 | | 4 |
| 9 | | 8 |
| 1 | | 14 |
| 2 | | 10 |
| 4 | | 6 |
| 12 | | 7 |
| 5 | | 6 |
| 23 | | 9 |
| 2 | | 11 |
| 5 | | 8 |
| 8 | | 5 |
| 20 | | 7 |
| 5 | | 7 |
| 8 | | 4 |
| 2 | | 4 |
| 8 | | 6 |
| 5 | | 4 |
| 12 | | 7 |
| 3 | | 6 |
| 11 | | 6 |
| 14 | | 8 |
| 6 | | 12 |
| 2 | | 17 |
| 18 | | 8 |
| 18 | | 6 |
| 23 | | 4 |
| 4 | | 8 |
| Average | 16.52 | 7.75 |
| Standard deviation | 48.20 | 3.94 |
| Coefficient of variation | 2.91 | 0.50 |
