# Supplementary material for: Adaptation and Preadaptation of Salmonella enterica to Bile
Source: PLoS Genet. 2012 Jan 19;8(1):e1002459. doi: 10.1371/journal.pgen.1002459 (PMC3261920; doi:10.1371/journal.pgen.1002459)
Supplement: Table S5 — Oligonucleotides. (DOC) [file pgen.1002459.s006.doc]

**Table S5**. Oligonucleotides

| **Oligonucleotide name** | **Sequence (5’-3’)** |
| --- | --- |
| aroG-E1 | aacgcagcagagaatcttgc |
| aroG-E2 | caggaagtacctaagtacgc |
| aroG-P1-REV | cccgctttggtgaggccttctttcacctcagcaacatgctGTGTAGGCTGGAGCTGCTTC |
| aroG-P4-FOR | tctgctggtggtgatcggtccttgttcaattcatgatccgATTCCGGGGATCCGTCGACC |
| ecnB-E1 | ctggttcttcttaccagcac |
| ecnB-E2 | cacgcctttacatgagcttg |
| ecnB-P1-REV | gttgcagcgccagagatcgcactaccaccgtccgagatgtGTGTAGGCTGGAGCTGCTTC |
| ecnB-P4-FOR | gacaattgcagcgatcttttctgttttggtactttccactATTCCGGGGATCCGTCGACC |
| osmY-E1 | accgcattcagcaatgcaac |
| osmY-E2 | aatatgagcgctcaggctac |
| osmY-P1-FOR | actctgctggccgtaatgttgacctctgctgttgcgacagGTGTAGGCTGGAGCTGCTTC |
| osmY-P2-REV | acgccatcaacggctttcgcgatgctttcagcgcggtcgcCATATGAATATCCTCCTTAG |
| STM1441-E1 | caaagcgtggaaagtgactc |
| STM1441-E2 | cacattcatgccaacggtag |
| STM1441-P1-REV | tgcgagcagttcagcagcaccactccccaacgtaataaccGTGTAGGCTGGAGCTGCTTC |
| STM1441-P4-FOR | cgcgttcccgatggttaatattgtagaaatcacgcagttgATTCCGGGGATCCGTCGACC |
| STM1672-E1 | cagtactgtacggtgtagtg |
| STM1672-E2 | ctggtaatgcgctaaagcag |
| STM1672-P1-REV | gcaaaatgccgggtagcgctggcccagggcattcgcatcaGTGTAGGCTGGAGCTGCTTC |
| STM1672-P4-FOR | aatggtagcttgtttttcgtatatcgcacatgccagtaccATTCCGGGGATCCGTCGACC |
| ugpB-E1 | ctctctgtcgccttactatc |
| ugpB-E2 | ctttgcagcgaataccacag |
| ugpB-P1-REV | gcaccggggtttttatcgtaataaccctgctcgcgggtcaGTGTAGGCTGGAGCTGCTTC |
| ugpB-P4-FOR | caacgcgccggcaatattgcaggtttatgaggttgggacgATTCCGGGGATCCGTCGACC |
| yajI -E1 | gttcattacacaccaggcac |
| yajI -E2 | cgtaatcgattgcgttcacg |
| yajI -P1-REV | aacggtaacgaaatattgacatcactgggcgccagcaagcGTGTAGGCTGGAGCTGCTTC |
| yajI -P4-FOR | ccagatgcatcaaagcattagcaccctgaataaaagagatgATTCCGGGGATCCGTCGACC |
| ybjM-E1 | gtaacttgccaagacacagg |
| ybjM -E2 | cggcatgataacacctgttc |
| ybjM -P1-REV | aacgcacaccagaatacggcgctaaataaccacgcgagttGTGTAGGCTGGAGCTGCTTC |
| ybjM -P4-FOR | tatgcatgtacaaggcgcttttcgcgcggcagggcatcctATTCCGGGGATCCGTCGACC |
| yceK-E1 | gtttacactatcgggtctgg |
| yceK-E2 | tgtgcagcagttcaacgttc |
| yceK-P1-REV | agtagcagtgtatcgaagatcagtgagaagggcagatcgaGTGTAGGCTGGAGCTGCTTC |
| yceK-P4-FOR | gattaccctgagcggctgcggcagtattatcagcagaacgATTCCGGGGATCCGTCGACC |
| yiiU-E1 | gtttacactatcgggtctgg |
| yiiU-E2 | tgtgcagcagttcaacgttc |
| yiiU-P1-REV | agtagcagtgtatcgaagatcagtgagaagggcagatcgaGTGTAGGCTGGAGCTGCTTC |
| yiiU-P4-FOR | gattaccctgagcggctgcggcagtattatcagcagaacgATTCCGGGGATCCGTCGACC |
| ytfK-E1 | cgtaaggtcatggtcattcc |
| ytfK-E2 | ctaccgtatcgatcagttgc |
| ytfK-P1-REV | ataacctgacggttaacttcagacatcacagacaagtgctGTGTAGGCTGGAGCTGCTTC |
| ytfK-P4-FOR | taatactctgcaggagacaacaatgaaaattttccaacgcATTCCGGGGATCCGTCGACC |
| yrbK-FOR | gactatgtgacgcacattgc |
| yrbK-REV | ctcgtagtgcatatgggaag |
| acrD-P4-FOR | catttttgcctgggtgctggctatcctgttgtgtctgacaATTCCGGGGATCCGTCGACC |
| acrD-P1-REV | cgtatcagcacgaaaaacaggggtacaaagaagatagccaTGTAGGCTGGAGCTGCTTCG |
| acrD-E1 | ccaacaaggaagagagtcag |
| acrD-E2 | ttgaacgtgaactggggaac |
